# Increasing Trends in Mental Health Problems Among Urban Chinese Adolescents: Results From Repeated Cross-Sectional Data in Changsha 2016–2020

**DOI:** 10.3389/fpubh.2022.829674

**Published:** 2022-02-24

**Authors:** Zhipeng Wu, Biao Wang, Zhibiao Xiang, Zhulin Zou, Zhening Liu, Yicheng Long, Xudong Chen

**Affiliations:** ^1^National Clinical Research Center for Mental Disorders, Changsha, China; ^2^Department of Psychiatry, The Second Xiangya Hospital, Central South University, Changsha, China; ^3^The High School Attached to Hunan Normal University, Changsha, China; ^4^Bocai Experimental School, Changsha, China

**Keywords:** mental health, adolescents, depression, anxiety, sex difference

## Abstract

This study performed a repeated cross-sectional analysis to explore possible trends in mental health problems among Chinese adolescents over the years of 2016–2020. A total of 2,837 different seventh-grade students were surveyed in three waves from a junior high school in Changsha city, Hunan province in China (978 in 2016, 949 in 2019, and 910 in 2020) using the Mental Health Inventory of Middle School Students (MMHI-60). The results showed that obsessive-compulsive tendencies, interpersonal sensitivity, depression, anxiety, academic stress, and emotional disturbance problems were significantly increased in surveyed adolescents from 2016 to 2020. Moreover, positive rates of most of these problems were significantly higher in females than males, and were significantly increased in only females. These results highlight the importance of focusing on mental health problems among urban Chinese adolescents, especially among girls.

## Introduction

Childhood and early adolescence are crucial periods for mental health development with a particularly high risk for mental health problems (1, 2). These problems can persist into adulthood and even lead to serious mental diseases if they are undetected or not treated appropriately (3–5).

Over the past decades, China has witnessed great developments in economy and society (6). However, in contrast to the increase in economy, multiple studies have reported a dramatic decrease in mental health in China, especially in Chinese adolescents during the same period (6–9). For example, a recent cross-temporal meta-analysis showed that Chinese adolescents' depression was significantly increased from 1989 to 2018 (7). Another cross-temporal meta-analysis, which was based on data between 1992 and 2017, also reported that Chinese adolescents' anxiety level was significantly increased since 1992 (8). To improve public mental health, it is thus important to assess the prevalence and changes over time in the prevalence of mental health problems in Chinese adolescents (7).

Although having gained important insights into trends in mental health problems in Chinese adolescents in recent years, the above-mentioned published literatures are limited in several ways. First, almost all these studies were based on data before the year 2018, and reports on newly trends after that time point are sparse. Second, most of them only focused on a single dimension of mental health such as depression or anxiety symptoms, while a more comprehensive survey in multiple dimensions is rare. Third, most of these studies are cross-temporal meta-analyses, which pooled previous studies using the same mental health assessing instruments [e.g., Self-rating Depression Scale (7)] together. However, such an approach may not be able to fully address the potential bias caused by regional diversity [e.g., the pooled studies were across different provinces in China with different economic and educational levels (7)].

In this study, we performed a repeated cross-sectional analysis to overcome the above limitations. Repeated cross-sectional studies utilize the same instruments and methodologies in the same population over multiple time timepoints; therefore, they were thought to be most suitable for investigating trends of mental health statuses over time (10, 11). In specific, a total of 2,837 different urban adolescents from a sample high school in Changsha city, Hunan province in China were surveyed in three waves from 2016 to 2020. Mental health status was assessed using a validated multi-dimensional mental health scale, Mental Health Inventory of Middle School Students (MMHI-60) (12, 13) and compared between different years.

## Methods

This study uses data collected from 2016 to 2020 in a total of 2,837 different urban Chinese junior high school students in three waves (978 students in September 2016; 949 students in September 2019; and 910 students in September 2020). Note that all participants were new seventh-grade students when they completed the survey, and all students were from the same campus of a high school (Tianding campus of The High School Attached to Hunan Normal University and Bocai Experimental School in Changsha city, Hunan province, China). A total of 76 participants (46 males and 30 females) with missing data for any item of the MMHI-60 in the surveys have been excluded from the study; there was no significant difference in sex ratio or age between the excluded participants and included participants in any wave (Chi-square test or *t*-test, *p* > 0.05). All participants and their supervisors provided written informed consents and the study was approved by the Ethics Committees of the Second Xiangya Hospital of Central South University, Changsha (IRB Number: 2021015).

All participants completed the MMHI-60 in the classroom to assess their mental health statuses. MMHI-60 is a validated and widely used self-report measure of mental health problems, which includes 10 subscales of distinct dimensions (60 items in total, and 6 items for each subscale): obsessive-compulsive tendencies, paranoid ideation, hostility, interpersonal sensitivity, depression, anxiety, academic stress, maladaptation, emotional disturbance and psychological imbalance (12–16). All items were scored from 1 to 5, and higher scores indicate more severe mental health problems. According to Wang et al. (13), a cutoff of average points of a subscale ≥2 is identified as having a mental health problem (positive for that subscale). This cutoff has shown good specificity and sensitivity in previous research (14, 15, 17). Positive rates of each subscale were calculated based on such a cutoff.

Differences in the sex ratio of participants between different waves were compared using the Chi-square test. Referring to previously published studies (18, 19), the Cochran–Armitage trend tests were used to determine if there are increasing or decreasing linear time trends of all MMHI-60 subscales' positive rates from 2016 to 2020. Moreover, considering that sex differences in mental health among adolescents have been widely reported (11, 14, 20), positive rates of all subscales were further compared between males and females using the Chi-square test; the significances of time trends in positive rates were also tested in males and females separately using the Cochran–Armitage trend tests. All statistical significances were set at *p* < 0.05.

## Results

**Figure 1:**
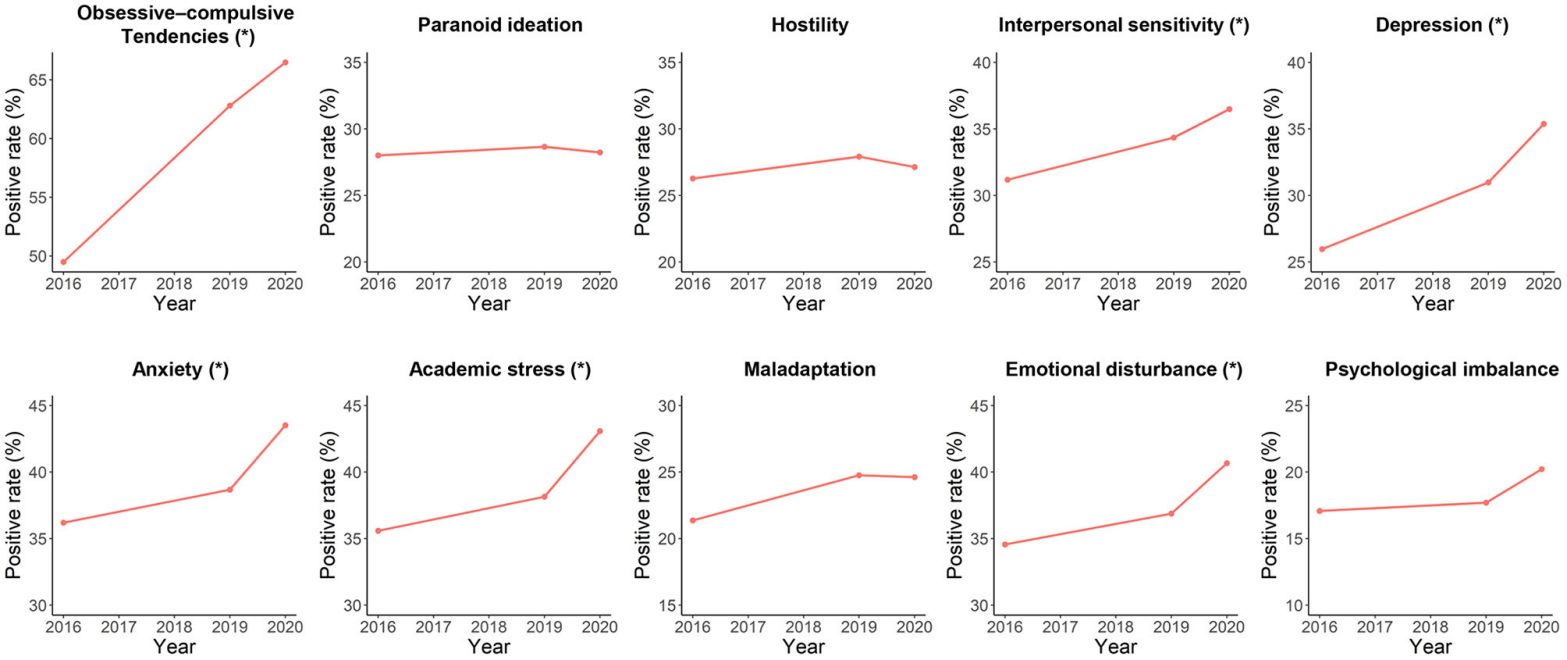
Trends in positive rates of each mental health problem in surveyed participants during the period of 2016–2020. The “*” indicates a significant increasing trend with *p* < 0.05.

Demographic and mental health characteristics of students in each wave were shown in Table 1. There was no significant difference in sex ratio among the three waves (χ^2^ = 1.873, *p* = 0.392). Data on participants' age was only available for the second wave and could not be compared. Cochran-Armitage trend tests showed that positive rates of the obsessive-compulsive tendencies (*p* < 0.001), interpersonal sensitivity (*p* = 0.015), depression (*p* < 0.001), anxiety (*p* = 0.001), academic stress (*p* < 0.001) and emotional disturbance (*p* = 0.006) problems were significantly increased during the period of 2016 to 2020, while no significant increasing or decreasing trends were found in positive rates of the paranoid ideation, hostility, maladaptation and psychological imbalance problems (all *p* > 0.05). The trends in positive rates of each mental health problem were also visualized in Figure 1.

**Table 1:** Sample characteristics of the participants surveyed in each wave.

	**Wave 1 (September 2016,** ***n* = 978)**	**Wave 2 (September 2019,** ***n* = 949)**	**Wave 3 (September 2020,** ***n* = 910)**	**Statistics**
Sex (male/female)	544/434	503/446	481/429	χ^2^ = 1.873, *p* = 0.392
Age (years)	Unavailable	12.027 ± 0.432	Unavailable	/
MMHI-60 results (positive/negative/positive rate)				
Obsessive–compulsive tendencies	484/494/49.5%	596/353/62.8%	605/305/66.5%	*z* = 7.566, *p* < 0.001
Paranoid ideation	274/704/28.0%	272/677/28.7%	257/653/28.2%	*z* = 0.115, *p* = 0.909
Hostility	257/721/26.3%	265/684/27.9%	247/663/27.1%	*z* = 0.437, *p* = 0.662
Interpersonal sensitivity	305/673/31.2%	326/623/34.4%	332/578/36.5%	*z* = 2.435, *p* = 0.015
Depression	254/724/26.0%	294/655/31.0%	322/588/35.4%	*z* = 4.437, *p* < 0.001
Anxiety	354/624/36.2%	367/582/38.7%	396/514/43.5%	*z* = 3.241, *p* = 0.001
Academic stress	348/630/35.6%	362/587/38.2%	392/518/43.1%	*z* = 3.326, *p* < 0.001
Maladaptation	209/769/21.4%	235/714/24.8%	224/686/24.6%	*z* = 1.683, *p* = 0.092
Emotional disturbance	338/640/34.6%	350/599/36.9%	370/540/40.7%	*z* = 2.731, *p* = 0.006
Psychological imbalance	167/811/17.1%	168/781/17.7%	184/726/20.2%	*z* = 1.753, *p* = 0.080

**Figure 2:**
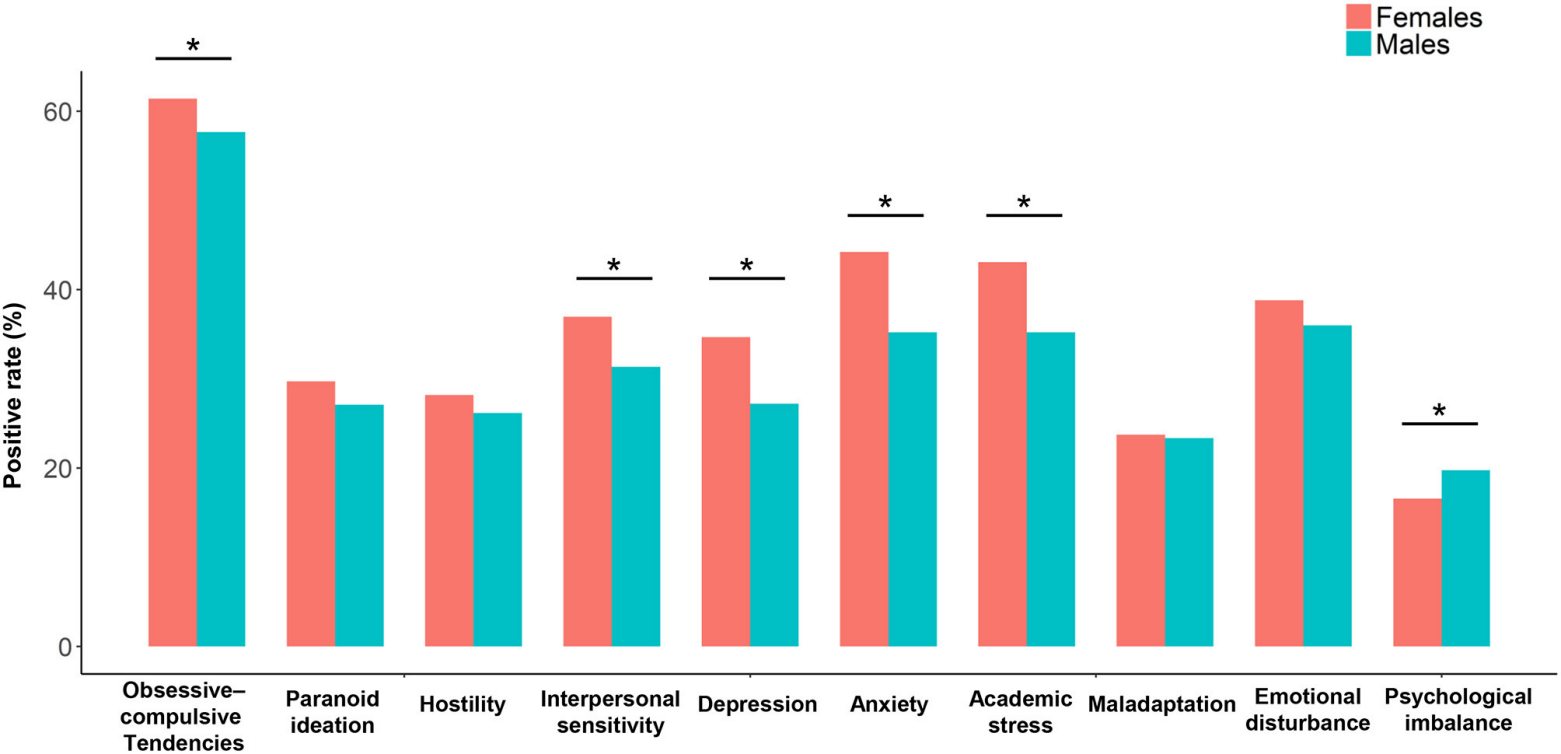
Comparisons on positive rates of each mental health problem between the female and male participants. The “*” indicates a significant sex difference with *p* < 0.05.

**Figure 3:**
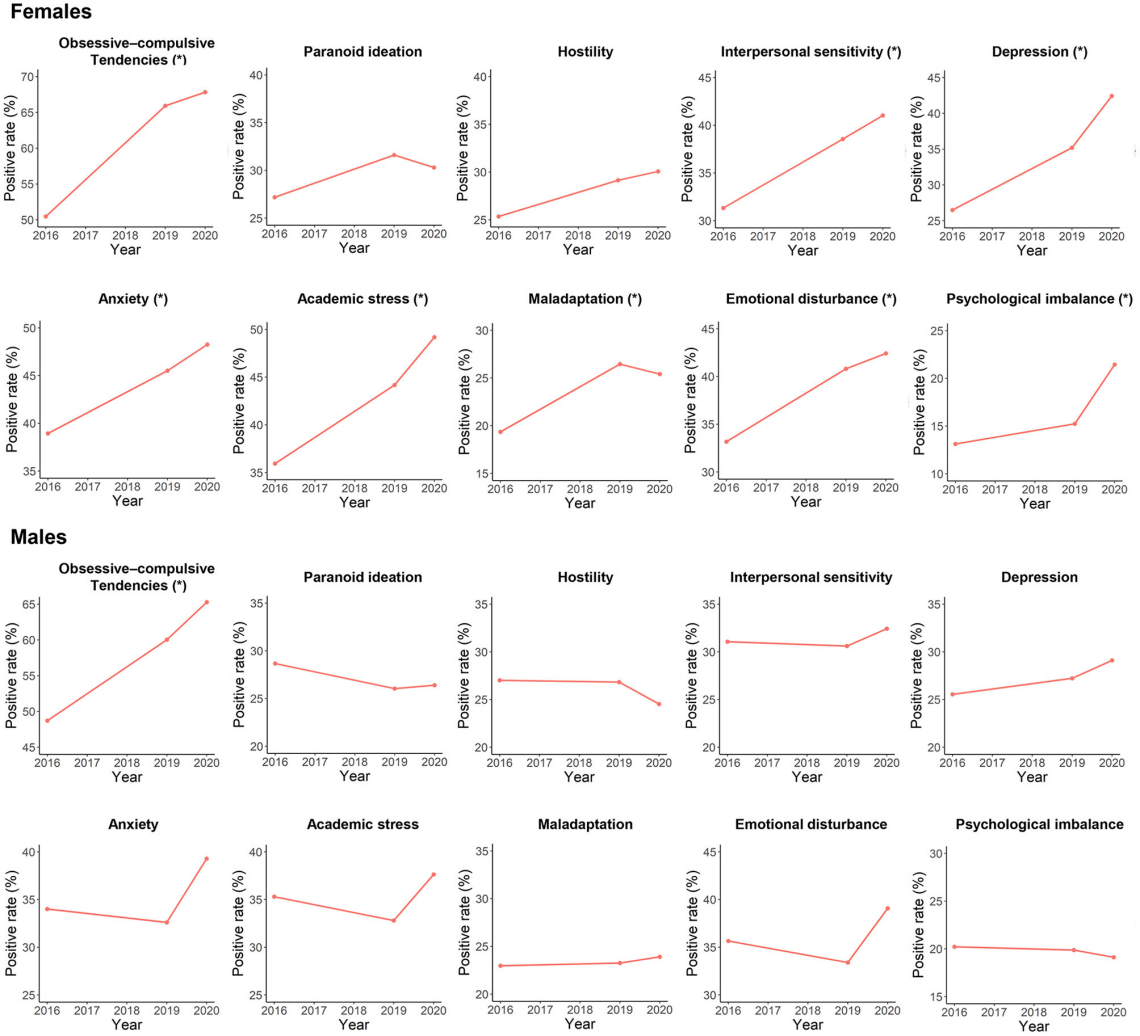
Trends in positive rates of each mental health problem during the period of 2016–2020 in surveyed females and males, respectively. The “*” indicates a significant increasing trend with *p* < 0.05.

The females had significantly higher positive rates of obsessive-compulsive tendencies, interpersonal sensitivity, depression, anxiety, and academic stress, but a significantly lower positive rate of psychological imbalance than males (Figure 2; Supplementary Table 1). From 2016 to 2020, the positive rate of obsessive-compulsive tendencies was significantly increased in both males and females, while positive rates of interpersonal sensitivity, depression, anxiety, academic stress, maladaptation, emotional disturbance, and psychological imbalance were significantly increased in only females (Figure 3; Supplementary Tables 2–3).

## Discussion

In this study, we performed a repeated cross-sectional analysis to explore the possible trends in mental health problems among Chinese adolescents over the years of 2016–2020. Compared with most published studies for similar purposes, we had advantages in methodologies (uniformity of surveyed populations) and data timeliness (until the most recent years).

Our results showed that positive rates of multiple mental health problems including obsessive-compulsive tendencies, interpersonal sensitivity, depression, anxiety, academic stress, and emotional disturbance problems were all significantly increased from September 2016 to September 2020 in surveyed participants (Figure 1). Some previous studies have reported similar trends of increasing mental health problems in Chinese adolescents; however, all these studies were based on data before the year 2018 (6–9). Thus, as a supplement to these studies, our study suggests the possibility that mental health in Chinese adolescents could have been deteriorating in the most recent years. The increases in mental health problems, as discussed in some previous studies, may be associated with the reported changes in several social and psychological factors in recent years in China [e.g., increasing divorce rate (21)]. It is noteworthy that the COVID-19 outbreak in 2020, which has been widely reported to have negative mental effects on the worldwide population (22–25). For instance, due to school closures and social isolation, adolescents may experience losses of close peer relationships during the pandemic which can lead to depression and anxiety (26, 27). Therefore, the pandemic-related effects may also play an important role in these trends besides other factors.

Further analyses showed that positive rates of most of the above mental health problems were significantly higher in females than males and were significantly increased in only females during 2016–2020. Although being somewhat surprising, similar results have been reported among adolescents in other countries that compared with boys, girls may be more affected by mental problems (28–30). There are multiple biological and social factors which might account for such sex differences. For example, adolescent girls are more likely than males to engage in social behaviors which exacerbate depressive symptoms in response to stresses (28, 31). Together, our results highlight the importance of focusing on mental health problems among urban Chinese adolescents, especially among girls.

It is notable that in our study, positive rates of some mental health problems were relatively high compared with results of several other studies in Chinese adolescents. For example, the prevalence of anxiety symptoms in 2020 was 43.5% in our sample, but only 37.4% (32) and 19% (26) in two other studies, respectively. Such difference may be partly attributed to the different scales used [e.g., the 7-item Generalized Anxiety Disorder scale to assess anxiety (26, 33)]. Using different scales may change the positive rates, which should be noted when comparing our results with those of other studies.

Our study has several limitations. First, this survey was not performed in the years 2017 and 2018. Second, data on age was lacking for most participants. However, since children were required to enter school until 6 years old in China (34), all participants should had a close age around 12 when they were surveyed. Third, we used Cochran-Armitage trend test based on the assumption that trends in all mental health problems are linear; however, the trends may also be non-linear. Lastly, the survey was performed in only a single school in an urban area (Changsha city), and only seventh-grade students in early adolescence. Thus, it should be cautious to interpret our results as trends in nationwide populations. Further studies conducted in a larger sample, in wider regions including rural areas, and in a wider range of age would be necessary to expand our knowledge in mental health among Chinese adolescents.

## Conclusion

In conclusion, in this study, we found that positive rates of multiple mental health problems were significantly increased during 2016–2020 in a group of surveyed adolescents in Changsha city, China. Moreover, positive rates of most of these problems were found to be significantly higher in females than males, and significantly increased during 2016–2020 in only females. Such results highlight the importance of focusing on mental health problems among Chinese adolescents, especially among girls.

## Data Availability Statement

The raw data supporting the conclusions of this article will be made available by the authors, without undue reservation.

## Ethics Statement

The studies involving human participants were reviewed and approved by Ethics Committee of the Second Xiangya Hospital of Central South University. Written informed consent to participate in this study was provided by the participants' legal guardian/next of kin.

## Author Contributions

ZW, XC, ZZ, and YL conceived the idea, designed the study, collected and analyzed the data, drafted, and approved the manuscript. All authors contributed to the article and approved the submitted version.

## Funding

This work was supported by the Natural Science Foundation of Hunan Province, China (2021JJ40851 to YL, 2020JJ5800 to XC) and the National Natural Science Foundation of China (82071506 to ZL).

## Conflict of Interest

The authors declare that the research was conducted in the absence of any commercial or financial relationships that could be construed as a potential conflict of interest.

## Publisher's Note

All claims expressed in this article are solely those of the authors and do not necessarily represent those of their affiliated organizations, or those of the publisher, the editors and the reviewers. Any product that may be evaluated in this article, or claim that may be made by its manufacturer, is not guaranteed or endorsed by the publisher.
